# Integrated analyses using RNA-Seq data reveal viral genomes, single nucleotide variations, the phylogenetic relationship, and recombination for *Apple stem grooving virus*

**DOI:** 10.1186/s12864-016-2994-6

**Published:** 2016-08-09

**Authors:** Yeonhwa Jo, Hoseong Choi, Sang-Min Kim, Sun-Lim Kim, Bong Choon Lee, Won Kyong Cho

**Affiliations:** 1 Department of Agricultural Biotechnology, College of Agriculture and Life Sciences, Seoul National University, Seoul, 151-921 Republic of Korea; 2 Crop Foundation Division, National Institute of Crop Science, RDA, Wanju, 55365 South Korea; 3 The Taejin Genome Institute, Gadam-gil 61, Hoeongseong, 25239 Republic of Korea

**Keywords:** Apple stem grooving virus, *De novo* genome assembly, Recombination, RNA-Seq, Single nucleotide variation, Transcriptome

## Abstract

**Background:**

Next-generation sequencing (NGS) provides many possibilities for plant virology research. In this study, we performed integrated analyses using plant transcriptome data for plant virus identification using *Apple stem grooving virus* (ASGV) as an exemplar virus. We used 15 publicly available transcriptome libraries from three different studies, two mRNA-Seq studies and a small RNA-Seq study.

**Results:**

We *de novo* assembled nearly complete genomes of ASGV isolates Fuji and Cuiguan from apple and pear transcriptomes, respectively, and identified single nucleotide variations (SNVs) of ASGV within the transcriptomes. We demonstrated the application of NGS raw data to confirm viral infections in the plant transcriptomes. In addition, we compared the usability of two *de novo* assemblers, Trinity and Velvet, for virus identification and genome assembly. A phylogenetic tree revealed that ASGV and *Citrus tatter leaf virus* (CTLV) are the same virus, which was divided into two clades. Recombination analyses identified six recombination events from 21 viral genomes.

**Conclusions:**

Taken together, our *in silico* analyses using NGS data provide a successful application of plant transcriptomes to reveal extensive information associated with viral genome assembly, SNVs, phylogenetic relationships, and genetic recombination.

**Electronic supplementary material:**

The online version of this article (doi:10.1186/s12864-016-2994-6) contains supplementary material, which is available to authorized users.

## Background

*Apple stem grooving virus* (ASGV) is a member of the genus *Capillovirus* in the family *Betaflexiviridae* [1, 2]. ASGV has been most commonly identified from apple, European pear, Japanese pear, and Citrus trees [3]. In addition, ASGV has been identified in lily [2] and kiwi [4], and it infects several virus indicator plants, including *Chenopodium*, *Cucumber*, *Nicotiana*, *Phaseolus*, and *Vigna* species [4]. ASGV infection in fruit trees is usually latent without disease symptoms [5]; however, ASGV sometimes causes serious viral diseases [6]. In many cases, fruit trees are co-infected by different viruses and viroids. For instance, apple trees showing fruit deformation, leaf deformation, and mosaic, chlorosis, and rusting symptoms in India were co-infected by *Apple chlorotic leaf spot virus* (ACLSV), *Apple mosaic virus* (ApMV), ASGV, *Apple stem pitting virus* (ASPV), and *Apple scar skin viroid* (ASSVd) [7].

The viral particles of ASGV are flexuous filaments 620–680 nm long and 12 nm wide [4]. ASGV has a single-stranded (ss) positive-sense monopartite RNA genome containing 5′ capping and a poly(A) tail at the 3′ region [2]. The genome size of ASGV is about 6,495 ~ 6,597 nucleotides (nt), and it encodes two overlapping open reading frames (ORFs), ORF1 (242 kDa) and ORF2 (36 kDa) [1, 2]. ORF1 encodes a polyprotein containing a replicase and coat protein (CP), while ORF2 encodes a movement protein (MP) that is overlapped with the replicase and CP regions [1, 2]. A previous study demonstrated that ASGV mutants with a stop codon between the replicase and CP coding regions were capable of systemic infection with decrease of pathogenicity [8]. This result revealed that expression of ASGV CP via a subgenomic RNA (sgRNA) was sufficient for viability of ASGV. Furthermore, mutational analysis revealed core promoter sequences required for the sgRNA transcription of ASGV and *Potato virus T*, which were conserved among viruses in the families *Alphaflexiviridae* and *Betaflexiviridae* [9].

Next-generation sequencing (NGS) produces huge amounts of sequencing data, which facilitate the identification of known and novel viruses and viroids in a wide range of plant species [10]. In addition, NGS can be applied in plant virus diagnostics [6, 11] and virus ecology [12]. Several types of NGS platforms—including HiSeq systems by Illumina, 454 FLX systems by Roche, and SOLiD systems by AB—have been developed [10]. Each NGS system has advantages and disadvantages [10]. The selection of proper NGS platforms is dependent on the purposes of the study. HiSeq systems produce high throughput with a relatively shorter read length, whereas 454 FLX systems generate low throughput with a longer read length. For example, the identification and diagnostics of known and novel viruses can be conducted by HiSeq systems [13], and viral genome sequencing using extracted viral RNAs can be performed by 454 FLX systems [12].

Moreover, NGS systems are useful for virus–host interaction studies. For instance, sRNA-Seq has been used for virus-derived siRNAs (vsiRNAs) of ASGV from ASGV-infected samples [14]. This study showed an increase in siRNA production towards the 3′ end of ASGV and several tRNA-derived sRNAs were differentially regulated by ASGV infection. A previous study identified 149 conserved and 141 novel miRNAs of pear associated with ASGV infection and found several miRNAs in response to high temperature, which was used to reduce ASGV titers in the shoot meristem tip [15]. Pear transcriptome analysis between ASGV-infected and ASGV-free apple samples has been conducted and identified 184 up-regulated and 136 down-regulated genes in ASGV infected shoot culture as compared to ASGV-free shoot culture [5].

Several approaches to detect ASGV have been developed, such as long-distance PCR (LD PCR) to amplify the complete genome of ASGV [16], multiplex reverse transcriptase (RT)-PCR for major apple viruses [17–19] and pear viruses [20], and immunochromatographic assays by monoclonal antibodies specific for CP [21]. Moreover, using available genome sequences for ASGV, two phylogenetic groups and four recombinants of 16 ASGV isolates have been identified [22], and the molecular evolution of subgenomic RNA of ASGV has been studied [1].

Several recent studies have demonstrated that many plant transcriptomes contain viral sequences that could be applied to studies associated with virus identification and viral genome assembly [23, 24].

In this study, we conducted *in silico* analyses using publicly available transcriptome data for viral genome assembly and identification using ASGV as an exemplar virus. We showed the application of transcriptome data for the analysis of single nucleotide variations (SNVs) on the ASGV genome. Moreover, the two viral genomes obtained were successfully applied in the phylogenetic and recombination analyses of known ASGV genomes.

## Results

### Identification and *de novo* assembly of ASGV genome from ASGV-infected apple mRNA transcriptome

Of the known viruses, we selected ASGV, which mostly infects fruit trees, including apple (*Malus domestica*) and pear (*Pyrus pyrifolia*). Due to the clonal propagation of fruit trees, the possibility of virus infection is very high. We screened several apple and pear transcriptomes and selected the transcriptomes infected by ASGV for further study (data not shown). Of the several previously reported apple transcriptomes in response to ASGV infection, we selected two studies: one that performed mRNA sequencing (mRNA-Seq) [5] and one that performed sRNA-Seq [25]. Both sets of samples included ASGV-infected and ASGV-free apple plants (Additional file 1).

**Fig. 1:**
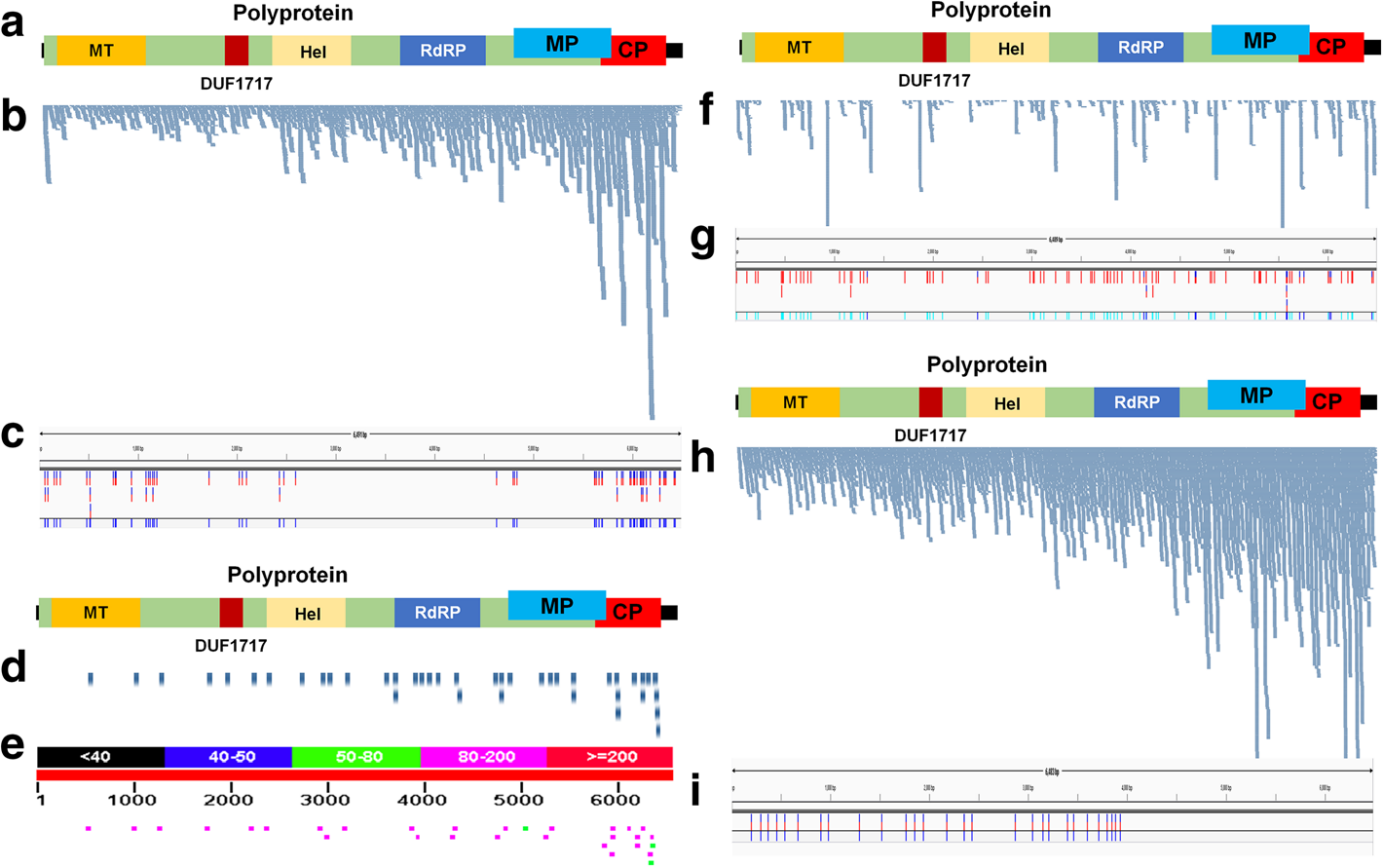
Identification of *de novo* viral genome assembly and SNVs for ASGV from RNA-Seq data. **a** Genome structure of ASGV isolate Fuji. The conserved domains were identified by the SMART program (http://smart.embl-heidelberg.de/). Abbreviations: MT (Methyltransferase), Hel (Helicase), RNA-dependent RNA polymerase (RdRP), Movement protein (MP), and Coat protein (CP). **b** Alignment of raw data against genome of ASGV isolate Fuji by BWA was visualized by Tablet program. **c** Positions of identified SNVs in ASGV-infected apple transcriptome were visualized by Tablet program. **d** Identified sequence reads from ASGV-free apple sample, which were associated with ASGV by BWA alignment. **e** BLAST results showing sequence reads from ASGV-free apple sample matched to ASGV genome. **f** Alignment of raw data using ASGV-infected sRNA data from cultivar GD against reference ASGV genome by BWA was visualized by Tablet program. **g** Positions of identified SNVs in ASGV-infected apple sRNA transcriptome were visualized by Tablet program. **h** Alignment of raw data from pear sample against genome ASGV isolate Cuiguan by BWA was visualized by Tablet program. **i** Positions of identified SNVs of ASGV in pear mRNA transcriptome were visualized by Tablet program

The first study was conducted to examine the expression profiles of apple trees infected with ASGV without any disease symptoms using mRNA-Seq [5]. Two libraries from ASGV-infected and ASGV-free shoot cultures were constructed. We *de novo* assembled transcriptomes of the two libraries using the Trinity program (Additional file 2). The 15,592 and 12,140 contigs obtained from ASGV-infected and ASGV-free shoot cultures, respectively, were blasted against a viral reference genome database. As a result, we identified 14 and 0 ASGV-associated contigs from ASGV-infected and ASGV-free samples, respectively (Table 1). Interestingly, the 14 identified ASGV-associated contigs mostly covered the complete genome of ASGV. Thus, we assembled a nearly complete ASGV genome with 6,454 nt. The newly assembled ASGV genome was referred to as ASGV isolate Fuji with the accession number KU500890. ASGV isolate Fuji contained two genes encoding the 241 kDa polyprotein and 36 kDa protein, respectively (Fig. 1a). The 241 kDa polyprotein is associated with viral methyltransferase, DUF1717, helicase, RNA-dependent RNA polymerase (RdRP), and the CP at the 3′ end, while the 36 kDa protein is known as a viral MP (Fig. 1a). The previous study sequenced the complete genome of ASGV (accession number KF434636) from the ASGV-infected sample by the Sanger sequencing method. To compare the ASGV genome sequences obtained by the Sanger method and *de novo* assembly, we aligned the two genome sequences by ClustalW. The two genome sequences were almost identical except the 3′ region, which showed many polymorphisms, indicating the presence of ASGV variants in the transcriptomes (Additional file 3).

**Table 1:** Blast results to identify ASGV-associated contigs from ASGV-infected apple mRNA-Seq data

Query id	Subject ids	Identity (%)	Alignment length	Mismatches	Gap opens	Query start	Query end	Subject start	Subject end	Evalue	Bit score
TR9237	NC_001749.2	83.29	1532	253	3	3	1533	12	1541	0	1408
TR10325	NC_001749.2	97.15	492	14	0	1	492	515	24	0	832
TR12482	NC_001749.2	98.1	473	9	0	1	473	945	473	0	824
TR1643|	NC_001749.2	97.71	1356	31	0	1	1356	1111	2466	0	2333
TR3149	NC_001749.2	98.28	232	4	0	1	232	1157	926	9.00E-113	407
TR1643	NC_001749.2	98.3	766	13	0	1	766	1701	2466	0	1343
TR9237	NC_001749.2	83.74	4705	741	23	1743	6435	1752	6444	0	4429
TR408	NC_001749.2	97.26	2225	61	0	1	2225	2440	4664	0	3771
TR8087	NC_001749.2	78.45	2237	452	30	1	2222	4660	2439	0	1434
TR2	NC_001749.2	83.26	723	119	2	1	722	5349	4628	0	664
TR9341	NC_001749.2	85.78	232	33	0	1	232	5389	5620	2.00E-64	246
TR5938	NC_001749.2	98.29	234	4	0	1	234	5647	5880	7.00E-114	411
TR12218	NC_001749.2	97.46	1065	27	0	1	1065	5683	4619	0	1818
TR9237	NC_001749.2	95.6	432	19	0	1	432	6013	6444	0	693

The MEGABLAST results with the best hits were listed. Subject IDs indicates the identity number of an individual assembled contig. Subject ids indicate the best matched viral genome

It is well known that RNA viruses have a quasispecies nature with a high mutation rate within infected hosts. Thus, we analyzed the SNVs of ASGV in the ASGV-infected sample. We mapped raw data on the genome of ASGV isolate Fuji, and interestingly, reads were highly mapped on the regions for CP and MP (Fig. 1b). Using the SAMtools program, we identified 90 SNVs. In particular, many SNVs were identified in the 5′ and 3′ regions of the ASGV genome (Fig. 1c and Additional file 4).

In many previous studies, the assembled contigs or transcripts were frequently used to identify viruses or viroids in the host transcriptome [26]. Although the assembled contigs did not contain any viral sequences in the ASGV-free sample, it is possible that the raw sequence data contained viral sequences. The single- or paired-end mRNA sequencing by HiSeq2000 produces raw sequence data up to 101 bp in size. Therefore, the raw data can also be successfully applied to identify viral sequences in the host transcriptome data. We aligned a raw FASTQ file from the ASGV-free sample on the genome of ASGV isolate Fuji using the BWA program. As shown in Fig. 1d, 41 sequenced reads were mapped on the genome of ASGV isolate Fuji. To confirm the alignment results, we blasted the FASTA converted sequences against the ASGV genome. We found that 30 sequenced reads were aligned along the ASGV genome (Fig. 1e). The mapping and blast results using sequenced raw data clearly demonstrated the presence of ASGV viral sequences in the ASGV-free sample.

### Identification and *de novo* genome assembly of ASGV from ASGV-infected sRNA transcriptomes

Previous studies have demonstrated that both mRNA-Seq and sRNA-Seq are useful for virus identification [26, 27]. To validate the utility of sRNA-Seq data for the *de novo* assembly of the ASGV genome, we used sRNA data from a previous study that conducted apple leaf sRNA sequencing using samples from the apple cultivar Golden Delicious (GD) [25]. The data were composed of 12 libraries from ASGV-infected and ASGV-free samples (Additional file 1). Moreover, two different types of libraries were generated according to size fraction [25].

The six libraries from ASGV-infected samples were subjected to *de novo* transcriptome assembly using the Trinity program followed by a blast search to identify viral contigs. However, we obtained only 209 contigs with 425 bp of N50 value, and no ASGV-associated contigs were identified by the blast search. It seems that the Trinity program was not optimal for *de novo* transcriptome assembly using sRNA data. Thus, we used the Velvet program, which is well known for sRNA transcriptome assembly [28]. The Velvet assembler assembled a total of 28,690 contigs, which were blasted against a plant viral database identifying 30 contigs associated with ASGV (Additional file 5). We mapped the identified ASGV-associated contigs on the reference genome of ASGV (NC_001749.2). The 30 contigs covered about 30 % of the ASGV genome and displayed many gaps along the genome. In order to confirm that sRNA reads cannot cover the complete genome of ASGV, we mapped sRNA raw data on the ASGV reference genome (Fig. 1f). We found that several regions of ASGV were not mapped by sRNA sequences. Based on the mapping results, we also identified 69 SNVs from sRNA data by the SAM Toolkit (Fig. 1g and Additional file 6).

### Identification and *de novo* genome assembly of ASGV from pear mRNA transcriptome

We used pear transcriptome data from a previous study that did not include any information on the virus infection. The transcriptome data (accession number SRX532394) was derived from a mixture of nine different fruit developmental stages of the *Pyrus pyrifolia* cultivar Cuiguan. The transcriptome was initially assembled by SOAPdenovo2; however, we performed *de novo* transcriptome assembly again using the Trinity program. A total of 33,858 transcripts were assembled (Additional file 7). Assembled sequences were subjected to a blast search against a viral reference database. We found nine contigs associated with ASGV ranging from 222 bp to 6,513 bp (Additional file 8). Of the nine contigs associated with ASGV, a single contig with 6,513 bp was a nearly complete genome sequence of ASGV. After removing poly(A) tails from the contig, we obtained a sequence with 6,488 nt referred to as ASGV isolate Cuiguan (accession number: KR185346).

In order to identify additional viruses infecting pears, all raw data converted to FASTA format were blasted against the viral reference database. Interestingly, we found many additional viruses infecting pears (Table 2). Of 11 viruses, six viruses including *Apricot latent virus*, *Grapevine fleck virus*, *Rupestris stem pitting associated virus-1*, ACLSV, *Grapevine Pinot gris virus,* and *Zucchini yellow mosaic virus* with very small numbers of reads were identified. Based on our knowledge, it seemed that the six identified viruses were not likely viruses infecting pears. They might have been sequences that were partially homologous to host genes or other viral genomes. In addition, associations of the six viruses with pears have not been reported. The sequence reads associated with *Potato leafroll virus* were identified as sequences from the host. Of four identified viruses infecting pears, ASGV was dominant followed by *Prunus virus T* (PrVT), *Apple green crinkle associated virus* (AGCAV), and ASPV.

**Table 2:** Identification of viruses from raw mRNA-Seq data of pear transcriptome by BLAST search

Name of virus	Accession no.	Size of genome	Read count
*Apple stem grooving virus*	NC_001749.2	6,495 bp	4274
*Potato leafroll virus*	NC_001747.1	5,987 bp	66
*Prunus virus T isolate Aze239*	NC_024686.1	6,835 bp	52
*Apple green crinkle associated virus*	NC_018714.1	9,266 bp	39
*Apple stem pitting virus*	NC_003462.2	9,332 bp	17
*Apricot latent virus*	NC_014821.1	9,311 bp	4
*Grapevine fleck virus*	NC_003347.1	7,564 bp	4
*Rupestris stem pitting associated virus-1*	NC_001948.1	8,744 bp	2
*Apple chlorotic leaf spot virus*	NC_001409.1	7,555 bp	1
*Grapevine Pinot gris virus*	NC_015782.1	7,275 bp	1
*Zucchini yellow mosaic virus*	NC_003224.1	9,591 bp	1

We examined SNVs for ASGV isolate Cuiguan within the pear transcriptome after alignment of the raw data on the ASGV isolate Cuiguan (Fig. 1h). We found 28 SNVs in the whole ASGV genome (Additional file 9). Interestingly, SNVs were only identified in the replicase region containing helicase, RdRP (Fig. 1i). However, SNVs were not detected in the region of MP or CP. Of the identified nucleotide changes, C to T (10 SNVs) was dominant followed by T to C (6 SNVs), G to A (6 SNVs), and A to G (6 SNVs).

### Comparison of *de novo* sequence assemblers for viral genome assembly

**Fig. 2:**
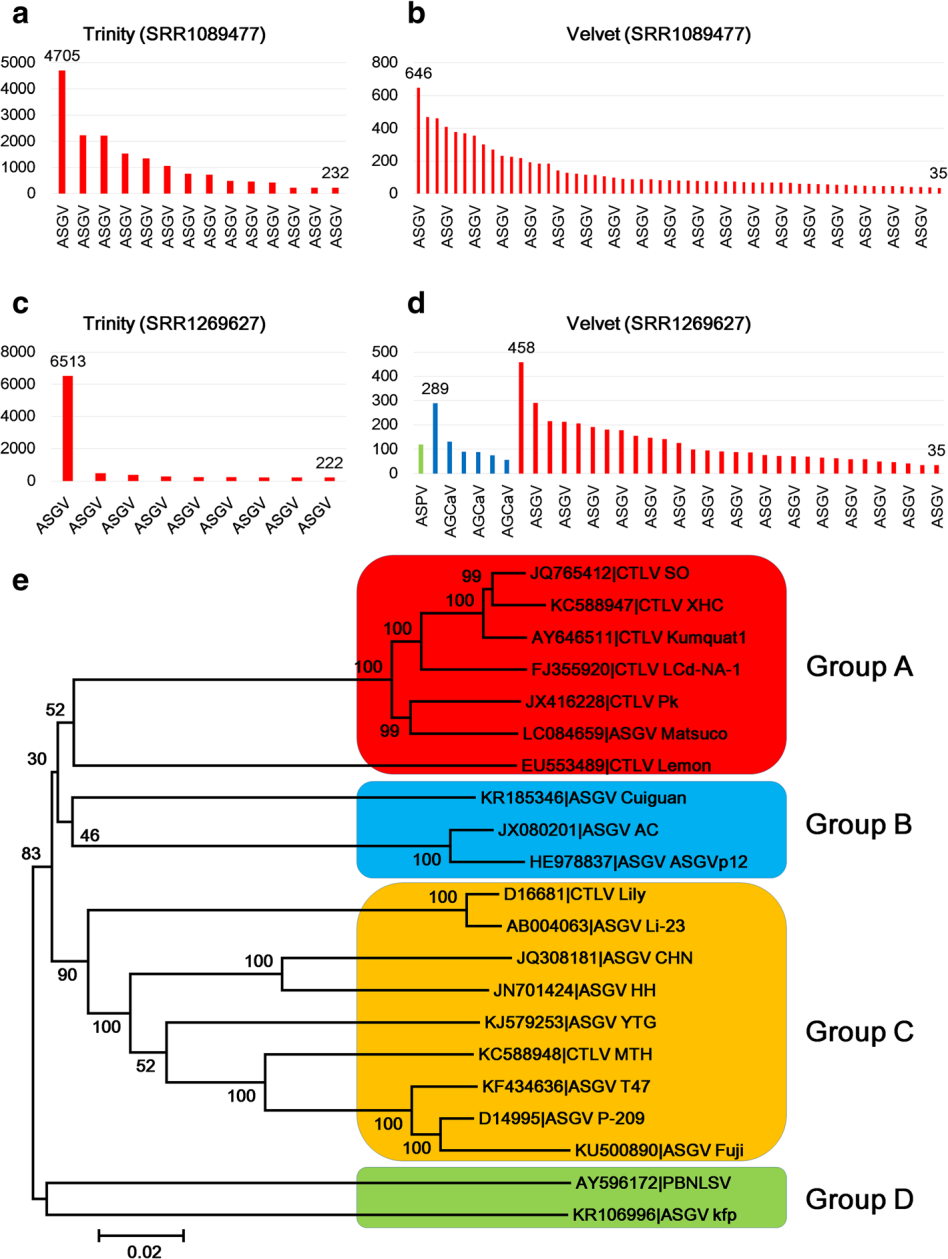
Comparison of two *de novo* assemblers based on number and sizes of assembled contigs and phylogenetic tree of 21 ASGV isolates. Size distribution of identified viral contigs from ASGV-infected apple sample assembled by Trinity (**a**) and Velvet (**b**). Size distribution of identified viral contigs from pear sample assembled by Trinity (**c**) and Velvet (**d**). The green-, blue-, and red-colored bars indicate ASPV, AGCAV, and ASGV, respectively. The sizes of only the longest and the shortest contigs in each transcriptome are indicated. (**e**) The phylogenetic tree was constructed based on the genome sequences of 13 ASGV isolates and 8 CTLV isolates. We followed the original annotations for CTLV and PBNLSV, which were highly homologous to ASGV. The accession number and the name of each isolate were indicated. Detailed information for each isolate can be found in Additional file 10

In this study, we used two different programs for *de novo* transcriptome assembly, Trinity and Velvet. To find the advantages and disadvantages of the two programs, we compared the number of total contigs and sizes of viral contigs. We first compared the number of contigs of two different mRNA libraries assembled by the two programs (Table 3). The number of contigs assembled by Velvet was more than 5.7 to 23.8 times that assembled by Trinity (Table 3). In addition, the number of identified viral contigs by Velvet was more than four times that identified by Trinity. However, the portion of viral contigs in the transcriptome assembled by Trinity was higher than that assembled by Velvet (Table 3). Moreover, the viral contigs assembled by Trinity were much bigger than those assembled by Velvet (Figs. 2a–2d). As a result, the Velvet assembler assembled large numbers of contigs with relatively short lengths, while the Trinity assembler assembled a few contigs with relatively long lengths. For example, in the transcriptome from the SRR1089477, the longest contigs assembled by Trinity were 4,705 bp, while the longest contigs assembled by Velvet were 646 bp (Figs. 2a and 2b). Furthermore, the Velvet assembler assembled seven contigs associated with ASPV and AGCAV that were not assembled by Trinity (Fig. 2c and 2d).

**Table 3:** Comparison of *de novo* transcriptome assemblers for assembly of viral contigs

	Trinity (SRR1089477)	Velvet (SRR1089477)	Trinity (SRR1269627)	Velvet (SRR1269627)
No. of total contigs	33858	195149	15592	371745
No. of viral contigs	14	57	9	37
% of viral contigs	0.04	0.03	0.06	0.01

SRR1089477 and SRR1269627 were data derived from pear and apple samples, respectively

### Phylogenetic analysis of ASGV isolates

Several previous studies have reported that ASGV is closely related to *citrus tatter leaf virus* (CTLV) [29]. To confirm previous results, we blast identified two ASGV genomes in this study against the NCBI nucleotide database. The blast results confirmed that CTLV is closely grouped with ASGV isolates in the genus *Capillovirus*. From the GenBank, we retrieved all ASGV-associated sequences as well as CTLV-associated sequences. After removing partial sequences, we collected a total of 21 genomes of ASGV and CTLV isolates, including two ASGV isolates in this study. The host ranges of CTLV were mostly from *Citrus* species as well as *Lilium* species (Additional file 10). Pear black necrotic leaf spot virus (PBNLSV) isolated from pear was an isolate of ASGV according to the annotation in GenBank [30]. Most ASGV isolates were isolated from apple and pear, and some isolates, such as ASGV isolates Matsuco and Li-23, were identified from *Citrus tamurana* and Lily, respectively. To reveal phylogenetic relationships, we aligned genome sequences displaying high sequence similarity of ASGV and CTLV. Sequence alignment and a phylogenetic tree using genome sequences of ASGV and CTLV identified two largely divided clades (Fig. 2e). The first clade contained 19 genomes, while the second clade included only PBNLSV and ASGV isolate KFP. The first clade could be further divided into three groups. Group A consisted of six CTLV isolates and a single ASGV isolate, while Group B contained only ASGV isolates. Group C was the largest, including seven ASGV isolates and two CTLV isolates.

### Recombination analysis for 21 ASGV isolates

**Fig. 3:**
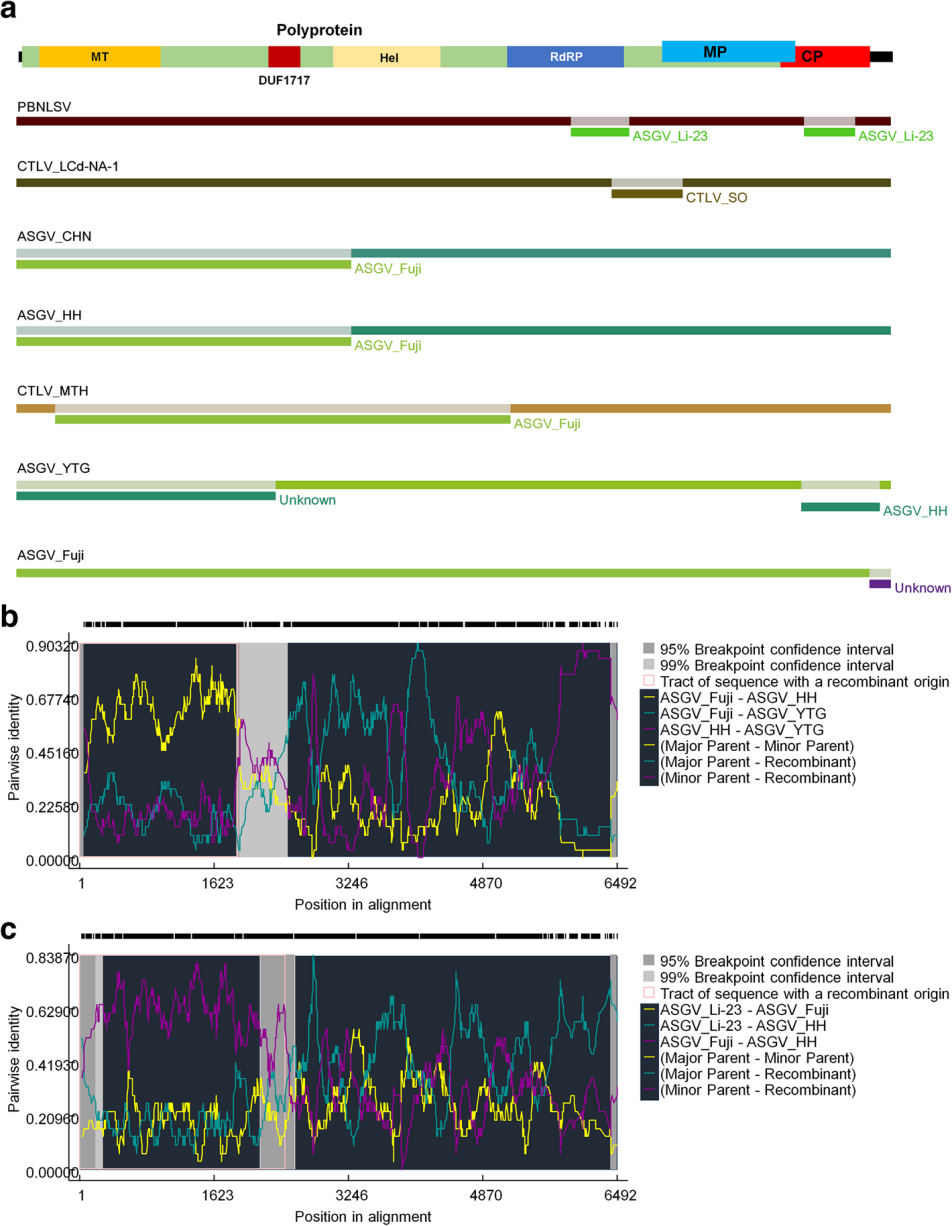
Identification of recombination events by RDP4 program. **a** The positions of identified recombinants were depicted with the respective names of parental sequences. The individual genome of the ASGV isolate was indicated by a different colored bar. The identified recombination events, including number 1 (**b**) and number 2 (**c**), were rechecked by the RDP4 program and visualized by plot data with pairwise identity information. Detailed information on the identified recombination events is provided in Table 4

We analyzed recombination events among 21 ASGV isolates. The aligned genome sequences were subjected to the RDP4 program, which includes nine different algorithms for recombination detection. The RDP4 program detected a total of 25 recombination events. Of them, we selected six recombination events that were supported by at least five recombination algorithms (Fig. 3a and Table 4). For example, PBNLSV contains two recombination sequences from ASGV isolate Li-23. Three isolates—ASGV isolate CHN, ASGV isolate HH, and CTLV isolate MTH—include recombination sequences from ASGV isolate Fuji in the 5′ region (Fig. 3a). Recombination Events 1 and 2 were supported by seven algorithms. The major parent of the recombinant sequence for ASGV isolate YTG was ASGV isolate Fuji (Fig. 3b). The major parent of the recombinant sequences for the three isolates—ASGV isolate HH, ASGV isolate CHN, and CTLV isolate MTH—were ASGV isolate Li-23 and CTLV isolate Lily (Fig. 3c).

**Table 4:** Recombination analysis of 21 ASGV genomes using RDP4 program

	In alignment		In recombinant sequence		Relative to CTLV_Lily					Detection methods								
Event Number	Begin	End	Begin	End	Begin	End	Recombinant sequence(s)	Minor parental sequence(s)	Major parental sequence(s)	RDP	GENECONV	Bootscan	Maxchi	Chimaera	SiSscan	PhylPro	LARD	3Seq
1	6492	1926	6487	1924	6488	1925	ASGV_YTG	Unknown (ASGV_HH), Unknown (ASGV_CHN)	ASGV_Fuji	1.30E-31	3.39E-09	8.78E-30	1.16E-10	9.12E-16	9.16E-27	NS	NS	3.17E-17
2	6492	2488	6488	2487	6488	2487	ASGV_HH, ASGV_CHN, CTLV_MTH[P]	ASGV_Fuji, CTLV_MTH	ASGV_Li-23, CTLV_Lily	3.40E-19	3.89E-04	1.47E-16	1.29E-16	1.83E-10	1.06E-27	NS	NS	1.48E-13
3	4112	4552	4110	4550	4110	4550	PBNLSV	ASGV_Li-23, CTLV_Lily	ASGV_KFP	1.20E-12	2.22E-08	8.19E-11	2.20E-05	2.75E-05	1.41E-10	NS	NS	NS
4	5844	6224	5842	6221	5842	6221	PBNLSV	ASGV_Li-23, CTLV_Lily	Unknown (CTLV_Pk), Unknown (CTLV_Kumquat1), Unknown (CTLV_LCd-NA-1), Unknown (CTLV_SO), Unknown (CTLV_XHC), Unknown (ASGV_Matsuco)	6.90E-07	1.12E-08	1.61E-07	6.45E-04	6.44E-04	1.30E-06	NS	NS	NS
5	5819	6412^a^	5816	6408^a^	5817	6409^a^	ASGV_YTG	ASGV_HH, ASGV_CHN	ASGV_P-209, ASGV_T47	2.42E-08	2.14E-03	1.58E-09	2.89E-03	1.65E-02	1.97E-07	NS	NS	NS
6	4412	4946	4409	4943	4410	4944	CTLV_LCd-NA-1	CTLV_SO, CTLV_Kumquat1, CTLV_XHC	Unknown (ASGV_Matsuco)	1.20E-02	2.91E-04	1.01E-02	4.02E-04	5.76E-03	4.04E-03	NS	NS	NS

*Minor Parent* Parent contributing smaller fraction of sequence, *Major Parent* Parent contributing larger fraction of sequence, *Unknown* Only one parent and a recombinant need be in the alignment for a recombination event to be detectable. The sequence listed as unknown was used to infer the existence of a missing parental sequence, *NS* No significant P-value was recorded for this recombination event using this method

^a^The actual breakpoint position is undetermined (it was most likely overprinted by a subsequent recombination event)

## Discussion

The rapid development of NGS is enabling virologists to find viruses from numerous species [10, 31]. NGS-based approaches have identified not only known viruses but also novel viruses [32, 33]. In fact, many horticultural plants are frequently infected by viruses and viroids [11, 24, 34, 35]. In particular, fruit trees usually propagated by grafting and cuttage are reservoirs of various plant viruses and viroids [24, 34]. In addition, the big data produced by NGS techniques has prompted virus identification *in silico* [23, 24]. Here, we discussed the library types, sequencing methods, and *de novo* assembler for virus identification and viral genome assembly.

The majority of plant viruses are composed of RNA genomes, and DNA viruses also replicate via an RNA intermediate [36]. Thus, RNA-based transcriptome libraries are preferable to DNA-based genome libraries for virus identification. In the current study, we used published plant transcriptome data. To enrich viral RNAs, ribosome-deleted libraries are usually prepared using extracted total RNAs from virus-infected samples [37]. However, we demonstrated that the mRNA libraries using oligo d(T) were successfully applied for virus identification. Of course, RNA viruses with poly(A) tails such as ASGV are also easily identified by mRNA libraries. Similarly, several polyadenylated RNA viruses have been identified from sweet potato transcriptomes [38]. Several recent studies have also demonstrated that ribosome-deleted RNA libraries as well as plant mRNA libraries are suitable for the identification of viruses without poly(A) tails or viroids [23, 24, 39]. Therefore, it might be ideal to use ribosome-deleted libraries for studies only focused on viruses. In the case of studies of both viruses and host plants, mRNA libraries can be usefully applied [24].

In this study, we used data from two different library types, including mRNA and sRNA libraries that were single-end sequenced by the HiSeq2000 system. According to many recent studies, viral genomes have been *de novo* assembled from mRNA as well as sRNA data [24, 33]. In our study, we assembled nearly complete genomes of two ASGV isolates from the mRNA data; however, the sRNA data could cover only 30 % of the ASGV genome. We compared the numbers of sequencing reads between the mRNA and sRNA data. However, the numbers of sequence reads between mRNA and sRNA were very similar, indicating that the sequencing amount is not an important factor for viral genome assembly. In fact, when the number of sequencing reads is increased, the number of viral-associated reads is increased. Therefore, the quantity of the sequenced data might play an important role in *de novo* genome assembly. The number of pear (3,524,264,028 bases) transcriptomes was about ten times that of apple transcriptomes (364,090,972 bases). The sequence reads associated with ASGV were 7,668 viral reads out of 7,430,428 reads for the apple sample and 4,274 viral reads out of 97,896,223 reads for the pear sample. Although the number of total sequence reads in the apple sample was much smaller than that in the pear sample, the number of sequence reads associated with ASGV was about 1.8 times higher. This result suggests the amount of viral replication in the host might be also an important factor in *de novo* viral genome assembly. The portion of viral nucleic acids in the sample infected by virus is often low suggesting enrichment of virions prior to NGS [40]. For example, purification of double-stranded (ds) RNAs from the *Prunus* species followed by 454 pyrosequencing enabled to assemble four complete genomes of Asian prunus virus 1 (APV1), APV2, and APV3 [41]. This study demonstrated successful application of dsRNA purification for virus genome assembly using NGS technique.

In the case of sRNA, two different types were prepared based on size fraction [25]. The libraries without size fraction contain a large number of ASGV-associated reads, but the libraries with size fraction contain very few reads associated with ASGV. Of course, the sRNA libraries were targeted for the identification of viral sRNAs. We suppose that the small number of sRNAs might be related to the ability of the RNA silencing machinery in the host. In any case, a sufficient number of viral-associated reads is necessary for viral *de novo* genome assembly.

In addition, sequencing methods are important for virus identification and viral genome assembly. In this study, all transcriptome data were single-end sequenced by HiSeq2000. As compared to single-end sequencing, paired-end sequencing provides sequences from both ends of a fragment and generates high-quality and alignable sequence data. The advantages of paired-end sequencing have been previously reported [42]. Thus, paired-end sequencing was far superior for the identification and genome assembly of the target virus.

For virus identification, assembled contigs are frequently used. Therefore, the choice of *de novo* assembler affects the quality and quantity of virus identification. For instance, mRNA data were very efficiently assembled by Trinity; however, few and low-quality contigs were assembled from the sRNA data by Trinity. Our comparative studies between the two *de novo* assemblers suggest Trinity and Velvet for *de novo* assembly of mRNA data and sRNA data, respectively. The obtained viral contigs assembled by Trinity from mRNA data were low in number but long in length, while the viral contigs assembled by Velvet were high in number but short in length. For the *de novo* assembly of a target virus with high-quality mRNA data, Trinity is ideal. Velvet cannot assemble a nearly complete viral genome, but it assembled many contigs, which enabled us to identify additional viruses, for example, viruses in the pear transcriptomes. Recently, several programs IVA, PRICE, and VICUNA for *de novo* assembly of RNA virus genome have been developed [43–45]. The choice of optimal *de novo* assembler might be dependent on researchers and purposes.

It is well known that RNA viruses have a quasispecies nature within the host [46]. However, to date, most studies have shown the variants and mutation rates of target viruses using cloning-based Sanger sequencing methods [47]. In this study, we successfully demonstrated the usefulness of plant transcriptome data for revealing the SNVs of ASGV. In fact, it is quite difficult to find virus variants using transcriptome data, while cloning-based sequencing methods might reveal variants. However, the cloning-based approaches require a RT-PCR amplification procedure to amplify full-length viral genomes. Practically, the amplification of full-length viral genomes is not easy even though plant viruses are relatively small. We showed the presence of ASGV variants in the transcriptome by comparing the ASGV genome from the cultivar Fuji derived from the Sanger-sequencing method and *de novo* assembly. We did not judge which ASGV genome was the dominant ASGV genome; however, it is highly likely that the *de novo*-assembled ASGV was a consensus genome sequence of ASGV. The mutation rates of identified ASGV genomes were varied: 1.38 % (90 SNVs) in the Fuji, 1 % (69 SNVs) in the GD, and 0.43 % (28 SNVs) in the Cuiguan. We suppose that several factors—including hosts, viral replication, and environmental cues—might affect the mutation rates. The association of viral mutation rate with other factors will be an interesting subject for further study [48].

## Conclusions

Taken together, our study showed the successful application of plant transcriptome data for virus identification, viral genome assembly, and viral mutation rates. In addition, we discussed several factors, including library preparation, NGS systems, *de novo* assemblers, and sample conditions for virus identification and genome assembly.

## Methods

### Plant materials

Detailed information for plant materials can be found in the previous studies [5, 25]. In brief, RNA-Seq data were derived from three different plant materials including *Malus x domestica* cultivar Fuji (SRP034943), *M. x domestica* cv. Golden Delicious seedlings, grafted onto MM.109 rootstocks (SRP035543), and *Pyrus pyrifolia* cultivar Cuiguan (SRP041640).

### Raw data processing and de novo transcriptome assembly

In this study, we used RNA-Seq data from three different projects. The first study employed mRNA-Seq data composed of two libraries derived from ASGV-infected and ASGV-free apple samples [5]. The second study employed sRNA-Seq data composed of 12 libraries derived from ASGV-infected and ASGV-free apple samples [25]. The third study employed mRNA-Seq data composed of a single library from pear samples without information on the ASGV infection. Information on the plant materials and library preparation were described in detail in the previous studies. Detailed information on the raw data can be found in Additional file 1: Table S1. All data were single-end sequenced by HiSeq2000. All bioinformatics analyses were performed in the Linux (Linux Mint version 17) installed workstation (four 16-core CPUs and 256 GB ram). We downloaded raw data for 15 libraries with respective accession numbers from the sequence read archive (SRA) database using the SRA toolkit [49]. The raw SRA data were converted to FASTQ files using the SRA toolkit. For the *de novo* assembly of transcriptomes, we used two different programs, Trinity version 2.0.6 and Velvet version 1.2.10 [28, 50]. *De novo* transcriptome assembly was performed according to the manuals provided by developers with default parameters.

### Sequence mapping and identification of viral contigs

For sequence alignment on the reference viral genome, we used Burrows-Wheeler Aligner (BWA) software with default parameters [51] Standalone BLAST version 2.1.19 was installed in the Linux system. To identify viral sequences in the assembled contigs, we used MEGABLAST, which is optimized for highly similar sequences against complete reference sequences for viruses and viroids (http://www.ncbi.nlm.nih.gov/genome/viruses/) with Evalue 1e-5 as a cutoff. In addition, all raw data were converted to FASTA files using the SRA toolkit and subjected to a MEGABLAST search against the viral reference database with Evalue 1e-5 as a cutoff.

### *De novo* assembly of ASGV genomes

The viral contigs identified by the BLAST search were retrieved by the BLASTCMD program in the standalone BLAST system. To assemble ASGV genomes, the identified viral contigs were aligned against the ASGV reference genome (NC_001749.2) using ClustalW implemented in the MEGA6 program [52]. The nearly complete genome of ASGV was manually obtained. The poly(A) tail at the 3′ end of ASGV was removed. We obtained nearly complete genomes for ASGV isolate Fuji (accession number KU500890) and ASGV isolate Cuiguan (accession number KR185346) from apple and pear transcriptomes. In the case of ASGV isolate GD, the obtained contigs covered only 30 % of the ASGV complete genome. Therefore, ASGV genome isolate GD was not obtained by the *in silico* approach.

### Analysis of SNVs in transcriptomes

In order to analyze SNVs of ASGV genomes, the raw data were aligned on each identified viral genome using the BWA program with default parameters. In the case of ASGV isolate Fuji and ASGV isolate Cuiguan, the *de novo*-assembled genomes were used. For ASGV isolate GD, the ASGV reference genome sequence was used for alignment. The aligned SAM files by BWA were converted into BAM files by SAMtools [53]. For SNV calling, we sorted the BAM files and then generated the VCF file format using mpileup function of SAMtools [54]. BCFtools implemented in SAMtools was finally used to call SNVs. The positions of identified SNVs on the ASGV genome were visualized by the Tablet program [55].

### Phylogenetic and recombination analyses of ASGV genomes

To retrieve the ASGV genome sequences, we first retrieved all sequences related to ASGV from the nucleotide database in GenBank (http://www.ncbi.nlm.nih.gov/genbank/). After eliminating partial sequences, only complete or nearly complete genome sequences for ASGV and CTLV isolates were identified. A total of 21 genome sequences including two isolates in this study were aligned by the ClustalW program with default parameters. After alignment, we deleted unnecessary sequences and poly(A) tails at the 5′ and 3′ regions, respectively. The manually edited aligned sequences were subjected to the construction of a phylogenetic tree using the MEGA6 program. The phylogenetic tree was constructed by the neighbor-joining method with 1,000 bootstrap replicates and Kimura 2-parameter distance.

We used Recombination Detection Program (RDP) version 4.66 [31]. To identify recombinants in the 21 ASGV genomes, the sequences aligned by ClustalW were exported into MEGA file format using the MEGA6 program. We searched recombination events by nine different algorithms in the RDP4 program, and only recombination events supported by at least five algorithms were finally identified.

## Additional files

Additional file 1: Detailed information on RNA-Seq data used in this study. (XLSX 10 kb) Additional file 2: Summary of *de novo* transcriptome assembly for ASGV-infected and ASGV-free apple samples. We assembled raw data from two different libraries using the Trinity program. The statistics of assembled contigs were calculated by TrinityStats.pl in the Trinity program. (XLSX 8 kb) Additional file 3: Comparison of *de novo*-assembled genome and Sanger-sequenced genome for ASGV isolate Cuiguan. Genome sequences of ASGV isolate Cuiguan obtained from de novo assembly (KU500890) and Sanger sequencing (KF434636) were compared. The regions for RdRP were highly conserved between the two sequences, and the CP region showed SNVs indicated by red-colored characters. (TIF 782 kb) Additional file 4: SNVs of ASGV isolate Fuji in ASGV-infected sample. (XLSX 17 kb) Additional file 5: Blast result to identify ASGV-associated contigs assembled from ASGV-infected sRNA data. (XLSX 10 kb) Additional file 6: SNVs of ASGV isolate GD in ASGV-infected sample. (XLSX 17 kb) Additional file 7: Summary of *de novo* transcriptome assembly from the pear mRNA data. (XLSX 7 kb) Additional file 8: Blast result to identify ASGV-associated contigs from ASGV-infected pear mRNA-Seq data. (XLSX 8 kb) Additional file 9: SNVs of ASGV isolate Cuiguan in ASGV-infected sample. (XLSX 13 kb) Additional file 10: Detailed information of complete genome sequences for ASGV and CTLV isolates used for phylogenetic and recombination analyses. (XLSX 10 kb)

## References

[1] Liebenberg A, Moury B, Sabath N, Hell R, Kappis A, Jarausch W (2012). Molecular evolution of the genomic RNA of Apple stem grooving Capillovirus. J Mol Evol.

[2] Yoshikawa N, Sasaki E, Kato M, Takahashi T (1992). The nucleotide sequence of apple stem grooving capillovirus genome. Virology.

[3] Magome H, Yoshikawa N, Takahashi T, Ito T, Miyakawa T (1997). Molecular variability of the genomes of capilloviruses from apple, Japanese pear, European pear, and citrus trees. Phytopathology.

[4] Clover G, Pearson M, Elliott D, Tang Z, Smales T, Alexander B (2003). Characterization of a strain of Apple stem grooving virus in Actinidia chinensis from China. Plant Pathol.

[5] Chen S, Ye T, Hao L, Chen H, Wang S, Fan Z (2014). Infection of apple by apple stem grooving virus leads to extensive alterations in gene expression patterns but no disease symptoms. PLoS One.

[6] Massart S, Olmos A, Jijakli H, Candresse T (2014). Current impact and future directions of high throughput sequencing in plant virus diagnostics. Virus Res.

[7] Kumar S, Singh RM, Ram R, Badyal J, Hallan V, Zaidi A (2012). Determination of major viral and sub viral pathogens incidence in apple orchards in Himachal Pradesh. Indian J Virol.

[8] Hirata H, Yamaji Y, Komatsu K, Kagiwada S, Oshima K, Okano Y (2010). Pseudo-polyprotein translated from the full-length ORF1 of capillovirus is important for pathogenicity, but a truncated ORF1 protein without variable and CP regions is sufficient for replication. Virus Res.

[9] Komatsu K, Hirata H, Fukagawa T, Yamaji Y, Okano Y, Ishikawa K (2012). Infection of capilloviruses requires subgenomic RNAs whose transcription is controlled by promoter-like sequences conserved among flexiviruses. Virus Res.

[10] Barba M, Czosnek H, Hadidi A (2014). Historical perspective, development and applications of next-generation sequencing in plant virology. Viruses.

[11] Wu Q, Ding S, Zhang Y, Zhu S (2015). Identification of viruses and viroids by Next-Generation Sequencing and homology dependent and homology independent algorithms. Annu Rev Phytopathol.

[12] Roossinck MJ, Saha P, Wiley GB, Quan J, White JD, Lai H (2010). Ecogenomics: using massively parallel pyrosequencing to understand virus ecology. Mol Ecol.

[13] Kehoe MA, Coutts BA, Buirchell BJ, Jones RA (2014). Plant virology and next generation sequencing: experiences with a Potyvirus. PLoS One.

[14] Visser M, Maree HJ, Rees DJ, Burger JT (2014). High-throughput sequencing reveals small RNAs involved in ASGV infection. BMC Genomics.

[15] Liu J, Zhang X, Zhang F, Hong N, Wang G, Wang A (2015). Identification and characterization of microRNAs from in vitro-grown pear shoots infected with Apple stem grooving virus in response to high temperature using small RNA sequencing. BMC Genomics.

[16] Dhir S, Walia Y, Zaidi A, Hallan V (2015). A simplified strategy for studying the etiology of viral diseases: Apple stem grooving virus as a case study. J Virol Methods.

[17] Kumar S, Singh L, Ram R, Zaidi AA, Hallan V (2014). Simultaneous detection of major pome fruit viruses and a viroid. Indian J Microbiol.

[18] Ji Z, Zhao X, Duan H, Hu T, Wang S, Wang Y (2012). Multiplex RT-PCR detection and distribution of four apple viruses in China. Acta Virol.

[19] Hassan M, Myrta A, Polak J (2006). Simultaneous detection and identification of four pome fruit viruses by one-tube pentaplex RT-PCR. J Virol Methods.

[20] Yao B, Wang G, Ma X, Liu W, Tang H, Zhu H (2014). Simultaneous detection and differentiation of three viruses in pear plants by a multiplex RT-PCR. J Virol Methods.

[21] Kusano N, Iwanami T, Narahara K, Tanaka M (2014). Production of monoclonal antibodies specific for the recombinant viral coat protein of Apple stem grooving virus-citrus isolate and their application for a simple, rapid diagnosis by an immunochromatographic assay. J Virol Methods.

[22] Chen H, Chen S, Li Y, Ye T, Hao L, Fan Z (2013). Phylogenetic analysis and recombination events in full genome sequences of apple stem grooving virus. Acta Virol.

[23] Jo Y, Choi H, Yoon J-Y, Choi S-K, Cho WK (2016). In silico identification of Bell pepper endornavirus from pepper transcriptomes and their phylogenetic and recombination analyses. Gene.

[24] Jo Y, Choi H, Cho JK, Yoon J-Y, Choi S-K, Cho WK (2015). In silico approach to reveal viral populations in grapevine cultivar Tannat using transcriptome data. Sci Rep.

[25] Visser M, Van der Walt AP, Maree HJ, Rees DJG, Burger JT (2014). Extending the sRNAome of apple by next-generation sequencing. PLoS One.

[26] Li R, Gao S, Hernandez AG, Wechter WP, Fei Z, Ling K-S (2012). Deep sequencing of small RNAs in tomato for virus and viroid identification and strain differentiation. PLoS One.

[27] Seguin J, Rajeswaran R, Malpica-Lopez N, Martin RR, Kasschau K, Dolja VV (2014). De novo reconstruction of consensus master genomes of plant RNA and DNA viruses from siRNAs. PLoS One.

[28] Zerbino DR, Birney E (2008). Velvet: algorithms for de novo short read assembly using de Bruijn graphs. Genome Res.

[29] Yoshikawa N, Imaizumi M, Takahashi T, Inouye N (1993). Striking similarities between the nucleotide sequence and genome organization of citrus tatter leaf and apple stem grooving capilloviruses. J Gen Virol.

[30] Shim H, Min Y, Hong S, Kwon M, Kim D, Kim H (2004). Nucleotide sequences of a Korean isolate of apple stem grooving virus associated with black necrotic leaf spot disease on pear (Pyrus pyrifolia). Mol Cells.

[31] Roossinck MJ, Martin DP, Roumagnac P (2015). Plant virus metagenomics: Advances in virus discovery. Phytopathology.

[32] Al Rwahnih M, Daubert S, Golino D (2015). islas cm, Rowhani A. Comparison of next generation sequencing vs. biological indexing for the optimal detection of viral pathogens in Grapevine. Phytopathology.

[33] Kreuze JF, Perez A, Untiveros M, Quispe D, Fuentes S, Barker I (2009). Complete viral genome sequence and discovery of novel viruses by deep sequencing of small RNAs: a generic method for diagnosis, discovery and sequencing of viruses. Virology.

[34] Pallas V, Aparicio F, Herranz M, Amari K, Sanchez-Pina M, Myrta A (2012). Ilarviruses of Prunus spp.: A continued concern for fruit trees. Phytopathology.

[35] Koh KW, Lu H-C, Chan M-T (2014). Virus resistance in orchids. Plant Sci.

[36] SCHOLTHOF KBG, Adkins S, Czosnek H, Palukaitis P, Jacquot E, Hohn T (2011). Top 10 plant viruses in molecular plant pathology. Mol Plant Pathol.

[37] Marston DA, McElhinney LM, Ellis RJ, Horton DL, Wise EL, Leech SL (2013). Next generation sequencing of viral RNA genomes. BMC Genomics.

[38] Gu Y-H, Tao X, Lai X-J, Wang H-Y, Zhang Y-Z (2014). Exploring the polyadenylated RNA virome of sweet potato through high-throughput sequencing. PLoS One.

[39] Jo Y, Choi H, Yoon J-Y, Choi S-K, Cho WK (2015). De novo genome assembly of grapevine yellow speckle viroid 1 from a grapevine transcriptome. Genome Announc.

[40] Jensen RH, Mollerup S, Mourier T, Hansen TA, Fridholm H, Nielsen LP (2015). Target-dependent enrichment of virions determines the reduction of high-throughput sequencing in virus discovery. PLoS One.

[41] Marais A, Faure C, Candresse T (2016). New insights into Asian prunus viruses in the light of NGS-based full genome sequencing. PLoS One.

[42] Fullwood MJ, Wei C-L, Liu ET, Ruan Y (2009). Next-generation DNA sequencing of paired-end tags (PET) for transcriptome and genome analyses. Genome Res.

[43] Hunt M, Gall A, Ong SH, Brener J, Ferns B, Goulder P (2015). IVA: accurate de novo assembly of RNA virus genomes. Bioinformatics.

[44] Ruby JG, Bellare P, DeRisi JL (2013). PRICE: software for the targeted assembly of components of (Meta) genomic sequence data. G3 (Bethesda).

[45] Yang X, Charlebois P, Gnerre S, Coole MG, Lennon NJ, Levin JZ (2012). De novo assembly of highly diverse viral populations. BMC Genomics.

[46] Cuevas JM, Willemsen A, Hillung J, Zwart MP, Elena SF (2015). Temporal dynamics of intrahost molecular evolution for a plant RNA virus. Mol Biol Evol.

[47] Tromas N, Elena SF (2010). The rate and spectrum of spontaneous mutations in a plant RNA virus. Genetics.

[48] Duffy S, Shackelton LA, Holmes EC (2008). Rates of evolutionary change in viruses: patterns and determinants. Nat Rev Genet.

[49] Leinonen R, Sugawara H, Shumway M (2010). The sequence read archive. Nucleic Acids Res.

[50] Haas BJ, Papanicolaou A, Yassour M, Grabherr M, Blood PD, Bowden J (2013). De novo transcript sequence reconstruction from RNA-seq using the Trinity platform for reference generation and analysis. Nat Prot.

[51] Li H, Durbin R (2009). Fast and accurate short read alignment with Burrows–Wheeler transform. Bioinformatics.

[52] Tamura K, Stecher G, Peterson D, Filipski A, Kumar S (2013). MEGA6: molecular evolutionary genetics analysis version 6.0. Mol Biol Evol.

[53] Li H, Handsaker B, Wysoker A, Fennell T, Ruan J, Homer N (2009). The sequence alignment/map format and SAMtools. Bioinformatics.

[54] Danecek P, Auton A, Abecasis G, Albers CA, Banks E, DePristo MA (2011). The variant call format and VCFtools. Bioinformatics.

[55] Milne I, Bayer M, Cardle L, Shaw P, Stephen G, Wright F (2010). Tablet—next generation sequence assembly visualization. Bioinformatics.

