# Supplementary figures and images for: Integrated analyses using RNA-Seq data reveal viral genomes, single nucleotide variations, the phylogenetic relationship, and recombination for Apple stem grooving virus
Source: BMC Genomics. 2016 Aug 9;17:579. doi: 10.1186/s12864-016-2994-6 (PMC4977635; doi:10.1186/s12864-016-2994-6)

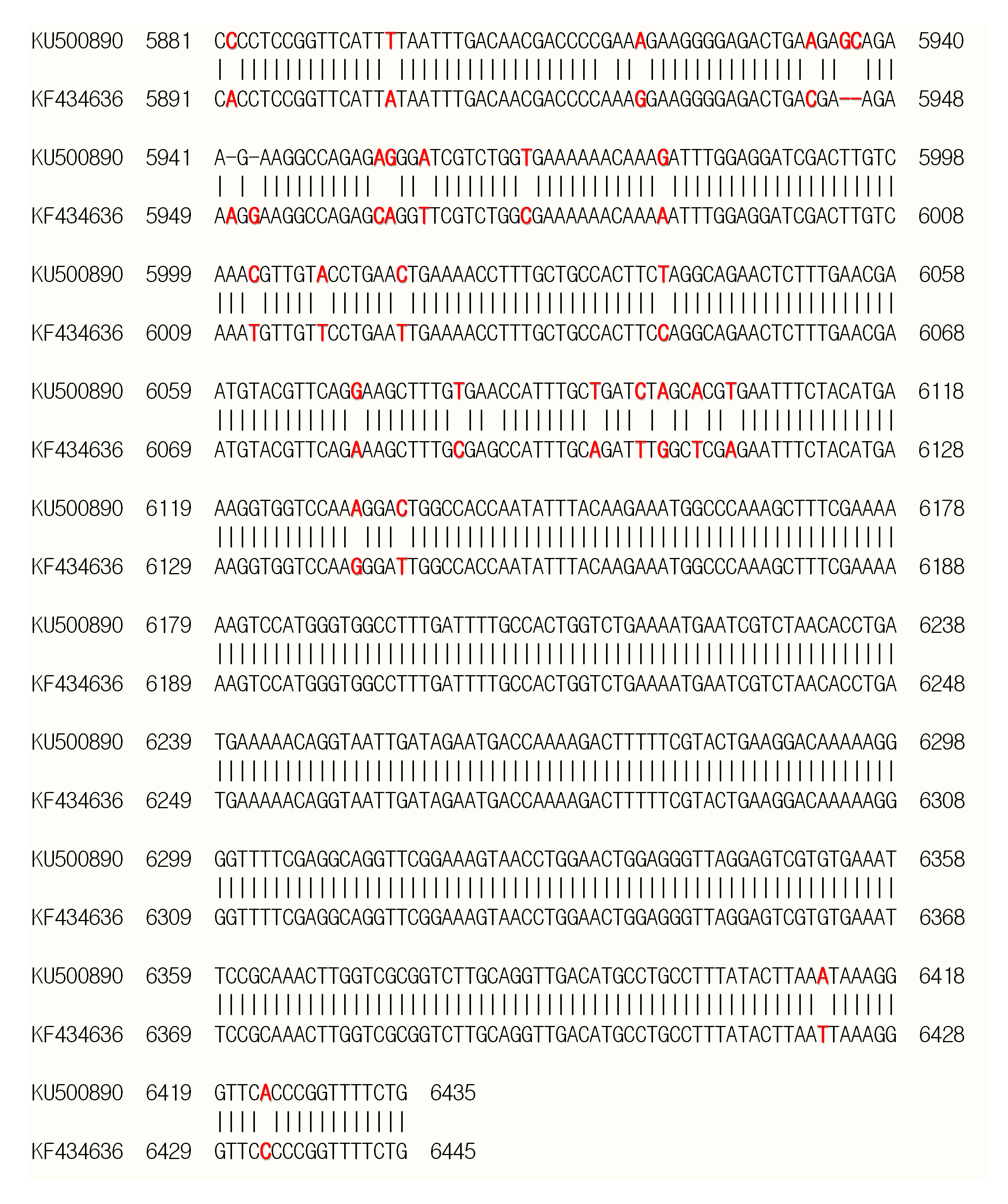

Supplement: Additional file 3: — Comparison of de novo-assembled genome and Sanger-sequenced genome for ASGV isolate Cuiguan. Genome sequences of ASGV isolate Cuiguan obtained from de novo assembly (KU500890) and Sanger sequencing (KF434636) were compared. The regions for RdRP were highly conserved between the two sequences, and the CP region showed SNVs indicated by red-colored characters. (TIF 782 kb) [file 12864_2016_2994_MOESM3_ESM.tif]
